# Why Are Acquired Search-Guiding Context Memories Resistant to Updating?

**DOI:** 10.3389/fpsyg.2021.650245

**Published:** 2021-03-01

**Authors:** Thomas Geyer, Werner Seitz, Artyom Zinchenko, Hermann J. Müller, Markus Conci

**Affiliations:** ^1^Department Psychologie, Ludwig-Maximilians-Universität München, Munich, Germany; ^2^Munich Center for Neurosciences – Brain & Mind, Ludwig-Maximilians-Universität München, Munich, Germany; ^3^Graduate School of Systemic Neurosciences, Ludwig-Maximilians-Universität München, Munich, Germany

**Keywords:** visual search, selective attention, contextual cueing, associative learning, memory systems, spike-timing dependent plasticity

## Abstract

Looking for goal-relevant objects in our various environments is one of the most ubiquitous tasks the human visual system has to accomplish (Wolfe, 1998). Visual search is guided by a number of separable selective-attention mechanisms that can be categorized as bottom-up driven – guidance by salient physical properties of the current stimuli – or top-down controlled – guidance by observers' “online” knowledge of search-critical object properties (e.g., Liesefeld and Müller, 2019). In addition, observers' expectations based on past experience also play also a significant role in goal-directed visual selection. Because sensory environments are typically stable, it is beneficial for the visual system to extract and learn the environmental regularities that are predictive of (the location of) the target stimulus. This perspective article is concerned with one of these predictive mechanisms: statistical context learning of consistent spatial patterns of target and distractor items in visual search. We review recent studies on context learning and its adaptability to incorporate consistent changes, with the aim to provide new directions to the study of processes involved in the acquisition of search-guiding context memories and their adaptation to consistent contextual changes – from a three-pronged, psychological, computational, and neurobiological perspective.

## Introduction

Extracting statistical regularities from a scene is a central capacity of the human visual system (e.g., Bar, 2004; Oliva and Torralba, 2007; Chetverikov et al., 2016; Hansmann-Roth et al., 2021). For example, if a searched-for target is repeatedly encountered in an invariant arrangement of distractor elements, observers can learn these configurations and use them to expedite search – an effect termed “contextual cueing” (Chun and Jiang, 1998). Contextual cueing is usually quite effective: it becomes evident after just a few repetitions and persists for at least a week (Chun and Jiang, 2003), with the underlying memory system exhibiting a rather high capacity (Jiang et al., 2005). However, context memory has also been shown to be severely limited (e.g., Manginelli and Pollmann, 2009; Makovski and Jiang, 2010; Conci et al., 2011; Zellin et al., 2011, 2013a,b, 2014; Conci and Müller, 2012; Annac et al., 2017). For instance, while observers are relatively quick to form long-term distractor-target memories for search guidance in an initial training phase, changes of the target location (in a subsequent test phase) within a repeated, that is, unchanged, distractor layout are difficult to incorporate within an established memory representation and adaptation to the change occurs only very slowly [see, e.g., Annac et al. (2017), for a representative meta-analysis of seven studies with *N* = 85 observers]. We have recently referred to this as the “down-side” of spatial context learning (Zinchenko et al., 2020a).

Here we consider possible reasons for the lack of adaptability of the contextual-cueing effect, focusing on the potential functional mechanisms, a related computational algorithm, and their possible neurobiological implementation. In a nutshell, from a neuro-cognitive perspective, we consider contextual cueing to reflect the ability to incidentally (effortlessly) extract and encode repeatedly encountered distractor-target relations in spatial long-term memory, the latter likely involving the medial temporal lobes (MTL). Once established, these memories are retrieved automatically by the search display presented on a given trial, with the activated contextual (distractor-to-target) associations providing pointers to the target location (and thus enhancing its activity) within the search-guiding attentional-priority map. Once consolidated, these representations come to generate memory-related costs in situations requiring contextual adaptation, that is, when the target is consistently repositioned to some other, “new” location within an otherwise unchanged distractor layout: in this case, the distractor context points to the “old” target location, thus cueing attention to the “wrong” location. Given that these memory-based (mis-) guidance signals are triggered automatically, it takes extensive practice to overcome them (cf. Shiffrin and Schneider, 1977). As we will argue below, this view lines in well with current theoretical and computational memory models that assume that contextual cueing is supported by relatively inflexible – that is, automatically acting – memory representations.

## A Functional Perspective Upon Context Adaptation

Cognitive theories of learning and memory play an important role for understanding contextual cueing and its resistance to adaptation in search tasks. For example, according to associative learning accounts (e.g., Rescorla and Wagner, 1972), frequent re-exposure to invariant distractor-target arrangements would strengthen the underlying representations of these items in memory. In this view, during the initial learning of repeated spatial layouts, associations are formed between the local, and/or global, distractor configuration and the target position (see, e.g., Brady and Chun, 2007; Shi et al., 2013; Beesley et al., 2015), and these learnt associations in turn facilitate search. However, associative learning can interfere with the acquisition of alternative associations in memory, a pattern referred to as associative blocking. Blocking occurs when a new cue is consistently paired with an outcome, given another cue has already proven to provide a valid predictor of that outcome (Kamin, 1968). As a result, the association between the second cue and the outcome is blocked. Applied to contextual-cueing in a training-phase/test-phase design, it is possible that the initially learned distractor-target associations dominate over potential associations incorporating the relocated target – hence, re-associating an “old” configuration with the changed target location will be blocked.

Blocking presupposes that the brain registers the changed target position in terms of some mismatch (or “prediction error”) signal between the current display input and the expected, that is, initially learned, target position. This mismatch signal would eventually also promote new learning, that is, the adaptation of contextual cueing to the changed distractor-target relations. Recent evidence suggests that the brain indeed works along these lines. For instance, in a recent study (Zinchenko et al., 2019), we applied repetitive transcranial magnetic stimulation (rTMS) over the left lateral frontopolar cortex (FPC), a structure known to be involved in the control of attention (Corbetta and Shulman, 2002). Stimulation of the FPC was compared to a posterior control site, and a no-rTMS baseline condition in a between-group manipulation, in order to examine how frontopolar cortex is involved in the updating of context-based memories subsequent to a target location change. The learning phase rendered reliable contextual cueing, with the cueing benefit being comparable across the three experimental groups. In the test phase, however, the recovery of cueing was critically dependent on stimulation site: while there was evidence of context adaptation toward the end of the experiment in the occipital- and no-rTMS groups, observers with FPC-rTMS showed no evidence of relearning whatsoever after target location changes. This finding shows that FPC plays an important role in both the representation and regulation of mismatch signals arising from changed target positions in repeated distractor layouts, suggesting that FPC is crucially involved in associative blocking.

Moreover, the degree to which blocking occurs might be dependent on the reliability of prediction errors (Friston, 2010; Hohwy, 2013), that is, their reliability determines whether errors are ignored or, respectively, used as learning signals for contextual adaptation (Friston, 2010; Hohwy, 2013). For instance, over the course of learning, observers may come to form predictions about regularities in the displays (or their absence) that then determine how a given search display is processed. For instance, a standard, “baseline” search experiment with typically (only) 50% repeated displays may not lead to the prediction that the sensory environment in which the system operates is “statistically rich.” As a result, learning may be turned off (Junge et al., 2007) – for instance, because contextual learning is resource-demanding (Annac et al., 2013; Travis et al., 2013). In terms of prediction errors, the turning-off of learning would be equivalent to the maintenance of the already established context memory “prior.”

That is, despite consistent repositioning of the target in the test phase, observers still exhibit a strong tendency to expect, and search for, the target at the initially learnt target location (see Manginelli and Pollmann, 2009; Zinchenko et al., 2020b, for relevant evidence).

Such an active-perception view of associative learning presupposes that contextual cueing will vary with sensory factors impacting on participants' implicit beliefs about contextual regularities in the current search environment, such as the relative proportion of repeated vs. non-repeated displays (signal vs. no-signal trials) or the rate of change between these displays (environmental stability/ volatility; e.g., Summerfield et al., 2011; Vaskevich et al., 2020). Having recently confirmed these predictions for initial context learning (Zinchenko et al., 2018), we are currently extending this approach to contextual re-learning.

In sum, associative learning models assume that a cueing effect develops for the initial target position, while responding to re-positioned targets should be slower and comparable to baseline, non-repeated, displays, due to the acquisition of the now-changed distractor-target regularities being blocked by the previously acquired contextual cues. Moreover, the relative strength of acquired context-target associations is determined by an observer's (implicit) “belief” about the general level of regularity prevailing in the current sensory environment, with potential implications for overcoming associative blocking and enabling contextual re-learning.

## The View From Computational Neuroscience

One promising computational mechanism that potentially mediates contextual cueing is spike-timing dependent plasticity (STDP; Markram et al., 2012; Goujon et al., 2015). A key feature of neurons equipped with STDP is that they are sensitive to repeated spike patterns (e.g., triggered by the repeated distractor-target arrangements). Accordingly, in a recent simulation (Seitz et al., 2021), we observed that a neural network equipped with STDP can effectively mimic human performance data in a contextual-cueing task. The network implemented by Seitz et al. (2021) consisted of over 2,000 neurons with an input and output layer and an additional hidden layer with sparse hidden-layer connectivity. Synaptic connections between the hidden layer and the output layer were learned with STDP. We analyzed the oculomotor scan-paths of human observers, which provide a potentially sensitive measure of the inspection – and importantly: the re-inspection on repeated encounters – of a given display (e.g., Noton and Stark, 1971). The analysis of human performance data showed the scan-paths to become more similar over successive viewings of a given repeated display. This finding was well-captured by a model equipped with STDP, but not by an alternative, control model without STDP. Thus, according to computational evidence, contextual cueing may be based on STDP mechanisms that change synaptic efficacies and thus equip neurons with the ability to become sensitive to repeated spatial patterns. However, once established, STDP-related memories may have a relatively long lifetime (e.g., Billings and van Rossum, 2009) and thus be resistant to updating.

## Context Adaptation From a Neurobiological Perspective

Despite the simplicity and persuasiveness of associative-learning models, considerations based on imaging and patient studies lend support to an alternative account of the (lacking) adaptability of contextual cueing. The relevant findings suggest that the relational memory underlying contextual cueing should in principle be flexible and thus be able to incorporate target position changes into previously acquired context memories. For instance, a bulk of neuroscientific data suggest that contextual cueing is supported by the medial temporal lobe (MTL), and especially the hippocampus (HC; e.g., Chun and Phelps, 1999; Greene et al., 2007; for review, see, e.g., Hannula and Greene, 2012), that is, memory structures that display representational flexibility in terms of the ability for re-combining information from past encounters with new events to adjust behavior (e.g., Wallenstein et al., 1998). However, these supramodal structures may work in tandem with other MTL structures, such as the parahippocampus (PHC; see Manns and Squire, 2001; Preston and Gabrieli, 2008; Geyer et al., 2012) – where PHC is usually considered as an automatic, rigid, or unitized memory system (Henke, 2010). Difficulties in adaptation might then be due to the relative dominance of one – inflexible – over the other – flexible – memory system in contextual cueing. Another possibility is that distractor-target associations are initially encoded in both the HC and PHC systems, along with the build-up of new (or the strengthening of existing) HC-PHC connections. Importantly, during frequent encounters of repeated sensory arrays, these connections might render inflexible PHC context memories independent of flexible HC memories [see, e.g., Frankland and Bontempi (2005), for this view, albeit in relation to other forms of – explicit – memory]. The latter idea also presupposes that the HC is only a temporary buffer and HC-buffered distractor-target associations are handed over to more durable, though inflexible, PHC memory structures, for instance, through memory consolidation during sleep (Geyer et al., 2013; Zellin et al., 2014).

Irrespective of the type of – HC and/or PHC – memory system supporting contextual cueing, a model of the memory-guided search must specify how and when context-based memories interact with basic visual-search processes. This question was addressed in a series of recent electrophysiological and patient studies showing that long-term memory for scenes aids visual processing very rapidly, with the earliest electrophysiological markers of guidance becoming evident only 80 ms post display onset at occipital electrodes contralateral to the hemifield of the target (Zinchenko et al., 2020b; see also Olson et al., 2001; Chaumon et al., 2008, 2009; Summerfield et al., 2011). Of note, Zinchenko et al. (2020b) found these early bias signals to persist even following consistent re-positioning of the target to another location within a previously learnt display layout. That is, learnt target positions can become very salient in search guidance, acting like an internal, memory-based (rather than an external) “singleton distractor” diverting attention away from the (now consistently changed) target location – a kind of induced “attentional-capture” effect (e.g., Theeuwes, 1992). The latter result is also important for understanding why contextual cueing is rather inflexible. Given that the existing memory triggers an automatic orienting response to the initially learnt target position, explicit awareness of the new target position (which would arise at the time of target selection) is only of limited effectiveness for unlearning the old position, thus delaying the incorporation of the changed position into the existing memory representation. This proposal refers back to “classical” work on associative learning that attributes a role to perceptual saliency of “attention” for explaining cue-competition effects [see, e.g., Kamin (1969), advocating that learning a novel cue is also a function of attention paid to that cue]. Automatic attraction of attention by learnt context cues also lines in well with reports suggesting that cueing manifests in the absence of explicit knowledge about the repeated displays (e.g., Colagiuri and Livesey, 2016; Spaak and de Lange, 2020; though see Kroell et al., 2019; Geyer et al., 2020, for explicit memory effects in contextual-cueing studies).

## Conclusions and Outlook

The phenomenon of contextual cueing illustrates that the human visual system constantly generates predictions from repeatedly encountered events and scenes in our environment that help us to rapidly detect and respond to critical target objects in familiar contexts (Chun and Jiang, 1998). However, changes of the target position in a previously learnt display array abolish the search (i.e., response-time) benefits rendered by repeated contexts, and facilitation for the new position recovers, or develops, only slowly, over the course of massive practice on the relocated displays (Zellin et al., 2013a,b, 2014). Here, we argue that the lacking adaptability of contextual cueing observed in training-phase/test-phase designs can be viewed at three distinct, though interrelated, levels of explanation: the functional perspective, that of (neuro-)computational theory, and that of the neurobiological implementation of contextual cueing. The functional view assumes associative learning mechanisms involving reinforcement and prediction: once established, a given distractor-target association exerts its influence and interferes with the build-up of novel associations by processes of associative blocking. Importantly, though, whether or not blocking occurs may be a function of the regularity prevailing in the sensory environment, i.e.,: observers' evaluation of (the reliability of) prediction errors and thus their eventual use of these signals for efficient context adaptation. Computational theory holds that STDP is the mechanism reliably implicated in statistical context learning in search tasks. Importantly, STDP-encoded memories would also be resistant to disruption, rendering the adaptation of cueing to changed contexts inefficient. The core assumption of the neurobiological view is the existence of both flexible and inflexible relational long-term memories and the contribution of both types of memory to the contextual-cueing effect (in various mixture ratios). Lack of adaptation could then result from the relative dominance of one (inflexible: PHC) over the other (flexible: HC) memory system during the re-learning phase.

We argue that, considered together, these perspectives can provide new impetus for research on the task factors that regulate the interplay of the two types of representation and the effects of more HC-dependent or more PHC-dependent representations on initial contextual learning and subsequent contextual adaptation (for first demonstrations, see, e.g.,: Lleras and Von Mühlenen, 2004; Annac et al., 2017; Higuchi and Saiki, 2017; Luque et al., 2017; Chen et al., 2019; Higuchi et al., 2019; Wang et al., 2020; Zinchenko et al., 2020a).

## Ethics Statement

The studies involving human participants were reviewed and approved by the Ethics Committee of the LMU Department Psychology. Participants provided their written informed consent to participate in this study.

## Author Contributions

TG, HM, and AZ conceived and designed the perspective article. WS and MC contributed to discussion. TG, HM, and MC wrote the paper. All authors contributed to the article and approved the submitted version.

## Conflict of Interest

The authors declare that the research was conducted in the absence of any commercial or financial relationships that could be construed as a potential conflict of interest.
